# Prevalence of elder abuse and victim-related risk factors during the COVID-19 pandemic in China

**DOI:** 10.1186/s12889-021-11175-z

**Published:** 2021-06-08

**Authors:** Pengcheng Du, Yu Chen

**Affiliations:** 1 Law School, Hunan University, Changsha, Hunan Province P.R. China; 2 School of Mathematics and Statistics, Hunan Normal University, Changsha, Hunan Province P.R. China

**Keywords:** COVID-19, Elder abuse, Risk factor, China

## Abstract

**Background:**

With the accelerated aging of the Chinese population, elder abuse has become a serious social problem. As COVID-19 has had a very large impact on economic development and lifestyle in China, it has also affected elder abuse. The purpose of this study is to estimate the prevalence of elder abuse in China during the COVID-19 pandemic, and to identify changes in risk factors for elder abuse in the context of COVID-19.

**Methods:**

We designed a cross-sectional study. In Hunan Province, a face-to-face questionnaire survey was conducted among elderly people over 65 years of age. To ensure the consistency of the measurement standards, we used the elder abuse questionnaire from the “Third Survey on Chinese Women’s Social Status.” According to related research, we selected 10 victim-related risk factors as independent variables. A logistic regression model was established to analyze the relationship between the independent variables and the four kinds of abuse.

**Results:**

We collected 10,362 samples from Hunan Province. During the COVID-19 pandemic, the prevalence of financial abuse and neglect was significantly higher than that in 2010. Income had a significant impact on the four types of abuse. The lower the income was, the greater the risk of abuse. Moreover, factors such as an older age, being a woman, a lower cognitive ability, and not having a cohabiting spouse increased the possibility of abuse. The greater the number of children was, the greater the risks of physical abuse, financial abuse, and elder neglect. Seniors with higher education levels, those who frequently participated in social activities, and those with religious beliefs were less likely to suffer abuse.

**Conclusions:**

During the COVID-19 epidemic, the prevalence of elder abuse in China has increased, which may be related to economic instability and social distancing measures. Increasing the income of the elderly and giving them more social support are important measures to reduce the prevalence of elder abuse.

## Background

Elder abuse has gradually become a global public health and human rights issue [1]. A meta-analysis from 28 countries showed that the prevalence of elder abuse is approximately 15.7%. The form of abuse with the highest prevalence was mental abuse at 11.6%, followed by financial abuse at 6.8%. The prevalence of neglect and physical abuse was 4.2 and 2.6%, respectively, and the form of abuse with the lowest prevalence was sexual abuse at 0.9% [2]. China is in a critical period of a transition to an aging society. According to the seventh census, individuals aged 60 and above account for 17.3% of China’s total population. It is estimated that the elderly population will increase to 280 million in 2025, accounting for 20% of the total population [3]. Elder abuse not only infringes on the rights of the elderly but also seriously affects their physical and mental health, increasing the risk of illness, and accelerating the death of elderly individuals [4].

In January 2020, coronavirus disease 2019 (COVID-19) broke out in China on a large scale. COVID-19 is a disease caused by infection with a new infectious respiratory virus. Although a variety of vaccines have been put into use, “social distancing” is still an important measure to prevent infection. The impact of social distancing is particularly problematic for the elderly because many elderly people normally face social isolation and loneliness [5]. Stress theory suggests that caring for the elderly is a difficult and stressful activity. When the work and life pressures faced by caregivers increase, they are likely to project these stresses onto elderly individuals, leading to an increase in the prevalence of abuse [6, 7]. COVID-19 has caused tremendous economic instability. A large number of workers have been fired or forced to take vacations, resulting in reductions in the income of adult family members and undoubtedly increasing financial pressures and psychological burdens within the family [8, 9]. The impact of COVID-19 on society, however, is even greater. Thus, elder abuse in China may have been affected. The purpose of this study is to estimate the prevalence of elder abuse in China during the COVID-19 pandemic, and to identify changes in risk factors for elder abuse in the context of COVID-19.

## The prevalence of elder abuse

In the United States, the prevalence of elder abuse differs depending on the race, health, and education level of the elderly individual. For elderly individuals with normal cognitive function the prevalence of elder abuse is 10% and for elderly individuals with dementia it is 47.3% [10]. Dong surveyed 3159 Chinese elderly individuals in Chicago and found that the prevalence of emotional abuse was 1.1% ~ 9.8%, that of physical abuse was 1.1%, that of sexual abuse was 0.2%, that of neglect was 4.6% ~ 11.1%, that of economic abuse was 8.8 to 9.3%, and the overall prevalence of elder abuse was 13. 9 to 25.8%. Abuse is more likely to occur in elderly individuals who are older, less educated, and in poor physical condition [11]. A household survey found that the overall prevalence of abuse among elderly low-income Latino immigrants in Los Angeles was 40.4%. Among these individuals, 25% experienced emotional abuse, 10.7% experienced physical abuse, 9% experienced sexual abuse, 16.7% experienced economic abuse, and 11.7% experienced neglect [12].

In Europe, the lowest prevalence of elder abuse is found in Ireland (2.2%), and the highest prevalence is found in Croatia (61.1%) [13, 14]. An epidemiological study that analyzed the entire United Kingdom found that the prevalence of elder abuse was 2.6%. The main form of abuse was neglect at 1.1%, followed by economic abuse at 0.6%, emotional abuse at 0.4%, physical abuse at 0.4%, and sexual abuse at 0.2% [15]. A large-scale epidemiological study conducted in Europe in 2009 administered surveys in 7 cities in 7 countries. Emotional abuse was the most common form of abuse (10.4% ~ 29.7%), followed by economic abuse (1.8% ~ 7.8%) and physical abuse (1.0% ~ 4.0%). Sexual abuse was the least common (0.3% ~ 1.5%) form of elder abuse. This result proves that the prevalence of elder abuse is varies between different countries [16].

In Africa, a semi-structured questionnaire survey of 404 elderly women in southwestern Nigeria revealed that 30% of elderly women had suffered abuse in the past year, the most common form being physical abuse [17]. A face-to-face survey of 1106 rural elderly individuals in Mansoura, Egypt, revealed that 43.7% of elderly individuals had been abused by their family members. The most common form of elder abuse was neglect at 42.4%, followed by physical abuse at 5.7%, and psychological abuse at 5.1%, and the least common form was economic abuse at 3.8%. Aging, an insufficient pension and having a caregiver other than a spouse are risk factors for elder abuse [18].

In Asia, a random survey about elder abuse was conducted in 7 states in India, and it was found that 11% of elderly individuals had experienced abuse. Among these individuals, 5.3% experienced physical abuse, 10.2% experienced verbal abuse, 5.4% experienced economic abuse, 6% experienced disrespect, and 5.2% experienced neglect. The main abusers were sons [19]. In South Korea, the prevalence of elder abuse is 6.3%. Experiencing abuse seems to be related to personal characteristics such as age, gender, education level, economic dependence, and physical health [20].

In China, Su Puyu conducted a survey of rural elderly individuals in Anhui Province and found that the reported rates of physical abuse, emotional abuse, economic abuse, and neglect were 6.0, 26.9, 4.9, and 7.2%, respectively. The main abusers were daughters-in-law and sons-in-law [21]. Wu Li et al. found that 36.2% of rural elderly individuals in Hubei Province were abused. Among these individuals, the prevalence rates of physical abuse, emotional abuse, neglect and economic abuse were 4.9, 27.3, 15.8, and 2.0%, respectively. Lack of social support and depression were found to be important factors in the occurrence of elderly abuse [22]. Research on elder abuse in the “Third Survey on Chinese Women’s Social Status” conducted by the Women’s Federation and the National Bureau of Statistics showed that the prevalence of elder abuse in China was 13.3%. The prevalence rates of physical abuse, emotional abuse, economic abuse, and neglect were 1.6, 4.9, 2.8, and 4.0%, respectively. The overall prevalence of elder abuse was 16.2% in rural areas and 9.3% in urban areas. The prevalence of elder abuse in rural areas was significantly higher than that in urban areas [23]. Differences in investigation methods and certification standards have a large impact on the results.

## Materials and methods

### Survey subjects

From April to May 2020, we conducted a questionnaire survey among elderly people aged 65 and above. People with language/communication impairments were excluded. We chose Changsha County, Ningxiang County, and Pingjiang County in Hunan Province as the survey locations. A total of 15 communities and 15 villages were randomly selected from each country. After obtaining the demographic information of the local elderly individuals over 65 years of age, we conducted a household questionnaire survey.

### Study design

This research is based on a cross-sectional design. The sample size was calculated using the formula: N = (Z_α/2_)^2^PQ / E^2^ (Z_α/2_, standard normal deviate at 95% confidence level; E, 1% relative error; P, prevalence = 50%; Q, 100% – prevalence). So the sample size should be at least 9604. Due to the entry conditions and the validity of the questionnaire, we needed to increase the number of survey subjects by 50%, so our sample size was 15,000 [21, 22]. A total of 15,000 questionnaires were distributed, and 10,362 valid questionnaires were recovered, with a recovery rate of 69.1%. A total of 5689 questionnaires were recovered from rural areas, and 4673 were recovered from urban areas. The survey takers were undergraduates majoring in epidemiology and health statistics who had undergone uniform and strict training. We communicated with each elderly individual alone in a room and told him/her that we would keep the conversation confidential. To prevent the spread of the epidemic, we wore masks. We also provided masks to the elderly individuals. A distance of 1 m was maintained during the conversation. To increase the likelihood of elderly individuals participating in the survey, daily necessities were distributed, and health consultations were performed. The questionnaire survey was conducted after obtaining verbal “informed consent” from the elderly individual.

### Variables and tools

The dependent variables of this study included physical abuse, emotional abuse, financial abuse, and neglect. The elderly abuse questionnaire in the “Third Survey on Chinese Women’s Social Status” is widely used to measure the prevalence of elder abuse in China (Cronbach’s alpha 0.81) [21, 23, 24]. To ensure the consistency of the measurement standards, we used this questionnaire. The investigator asked the elderly individual face-to-face, “Did your family exhibit any of the following behaviors towards you in the past year?” Eight behaviors that constitute domestic abuse were included in the survey: 1 Long-term refusal to visit, to greet, or speak to you; 2 Does not provide you with basic living expenses or embezzles your money privately; 3 Does not take care of you when needed; 4 Insults or threatens you; 5 Hits you; 6 Does not provide you with a fixed residence; 7 Does not feed you enough or feeds you poorly; 8 Does not allow you to leave the house. According to the aforementioned classifications, behaviors 5 and 8 were classified as physical abuse, behaviors 1 and 4 were classified as emotional abuse, behavior 2 was classified as financial abuse, and behaviors 3, 6, and 7 were classified as neglect. As long as one of the 8 behaviors was reported, the individual was considered to be abused, and a score of 1 was recorded; otherwise, a score of 0 was recorded.

According to related research, we selected the following 10 risk factors as independent variables. (1) Age: the actual age of the elderly individual [11, 22]. (2) Gender: 0 = male; 1 = female [22, 23]. (3) Marital status: 0 = married; 1 = divorced/widowed/unmarried [15]. (4) Educational level: 0 = illiterate; 1 = primary school to high school; 3 = university or above [21, 25]. (5) Income: 1 = under 5000; 2 = 5000 ~ 30,000; 3 = over 30,000 (RMB). If he or she is affected by COVID-19, the income of an elderly individual may decline [8, 9]. (6) Number of children: the number of living children. During the epidemic, the dependence of elderly individuals on their children increased [8, 22]. (7) Health condition (5-point system): 5 = very healthy; 1 = very unhealthy [18]. (8) Cognitive ability, or the basic cognition and calculation ability of the elderly individuals. This section mainly included questions such as “what day is today” and “how much is 30 minus 5.” There were a total of 30 questions in the survey. When an individual provided a correct answer, he or she was given 1 point. When he or she provided a wrong answers, he or she was not given any points. The lowest possible score was 0, and the highest possible score was 30 [26, 27]. (9) Social frequency: the frequency of participation in social activities over a week. If the elderly individual participated in social activities on a particular day, he or she was given a score of 1; otherwise he or she was given a score of 0. The lowest possible score was 0, and the highest possible score was 7. Less social participation frequency increases the risk of abuse, especially among men [28, 29]. During the epidemic, social distancing measures reduced the social participation frequency of the elderly [8, 9]. (10) Religious belief: 0 = yes; 1 = no [22, 23].

### Data analysis

In this study, SPSS 23.0 software was used for data input and analysis. First, the prevalence of elder abuse was analyzed. Then, the chi-square test was used to analyze the relationship between the sociodemographic characteristics of the elderly individuals and the prevalence of abuse. Finally, a logistic regression model was established to assess the relationship between the independent variables and the four kinds of abuse. The outcomes of the analysis were evaluated within a 95% confidence interval (CI), and statistical results with a *P*-value < 0.05 were considered significant.

## Results

### Prevalence of abuse among the elderly

**Fig. 1:**
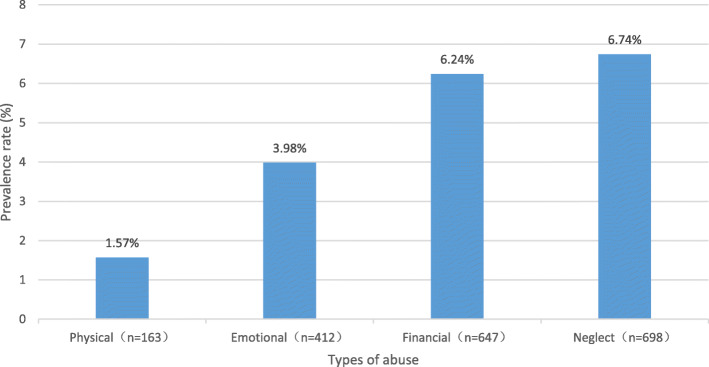
The prevalence of four types of elder abuse

This study revealed that the number of cases of abuse was 1596 and that the prevalence rate of elder abuse was 15.4%. Physical abuse (163) and emotional abuse (412) were less common than financial abuse (647) and neglect (698). A total of 31.2% of participants reported two or more types of abuse (Fig. 1).

### Factors associated with abuse

The study found that the prevalence of elder abuse increased with age, with the prevalence being 12.8% in the 65- to 69-year-old group, 16.2% on the 70- to 74-year-old group, and 21.1% in the 75-plus-year-old group. In terms of gender, women (16.7%) were more likely to be abused than men (13.7%). The prevalence of elder abuse among divorced, widowed, and unmarried individuals (20.0%) was higher than that among married individuals (13.3%). As the education level of the elderly individuals increased, the prevalence of abuse gradually decreased. The lower the income of an elderly person was, the higher the likelihood of abuse. The prevalence of abuse in the group earning more than RMB 30,000 was only 8.2%. The more children an elderly individual had, the higher the risk of abuse. The better the health and self-care ability of an elderly individual was, the lower the likelihood of abuse. The lower the cognitive ability of an elderly individual was, the higher the likelihood of abuse. The higher the social participation frequency of an elderly individual was, the lower the risk of abuse. Among elderly individuals with a weekly social frequency of 5–7, the prevalence rate of elder abuse was only 3.6%. People without religious beliefs (15.5%) were more likely to be abused than those with religious beliefs (11.0%). The chi-square test showed that the difference in the prevalence of abuse in each group was significant (*p* < 0.05) (Table 1).

**Table 1:** Relationship between sociodemographic characteristics and elder abuse

Characteristic		Total(n)	Abuse, n(%)	X^2^
Age	65 ~ 69	4559	584 (12.8)	61.77**
	70 ~ 74	4352	706 (16.2)	
	75~	1451	306 (21.1)	
Gender	Male	4446	608 (13.7)	19.78**
	Female	5916	998 (16.7)	
Marital status	Married	7119	947 (13.3)	76.99**
	Divorced	3243	649 (20.0)	
	Widowed			
	Unmarried			
Education level	Illiterate	6564	1092 (16.6)	44.44**
	Primary school to high school	3528	496 (14.1)	
	University and above	269	8 (3.0)	
Income	Under 5000	3232	584 (18.1)	49.86**
	5001 ~ 30,000	6349	948 (14.9)	
	30,001~	781	64 (8.2)	
Number of children	0	1039	112 (10.8)	28.58**
	1 ~ 2	2689	379 (14.1)	
	3~	6634	1105 (16.7)	
Health condition	1	2645	619 (23.4)	213.49**
	2 ~ 3	5936	835 (14.1)	
	4 ~ 5	1781	142 (8.0)	
Cognitive ability	0 ~ 10	243	63 (25.9)	65.37**
	11 ~ 20	2153	425 (19.7)	
	21 ~ 30	7966	1108 (13.9)	
Social frequency	0 ~ 1	9547	1541 (16.1)	51.58**
	2 ~ 4	731	52 (7.1)	
	5 ~ 7	84	3 (3.6)	
Religious belief	Yes	145	16 (11.0)	2.15*
	No	10,217	1580 (15.5)	

*p < 0.05, **p < 0.01

### Regression analysis

Age has a significant negative impact on the prevalence neglect, which means that the greater an individual’s age is, the greater the likelihood of being neglected. With increasing age, elderly individuals’ daily and psychological dependence on family members increases [30]. This imposes a very large burden or financial pressure on caregivers; thus the risk of elder neglect increases with age. Gender is significantly related to emotional abuse and neglect, as women are more likely to be emotionally abused and neglected [31]. The high prevalence of abuse among females may be related to the personality and psychological characteristics of females. In China, women are mostly housewives, and they depend financially on other family members. These women are in a disadvantaged position in the traditional sense and are relatively more vulnerable to abuse.

There is a significant correlation between income and the four types of abuse. The higher an individual’s income is, the less likely he or she is to be abused. Studies have found that the more financially well-of an elderly individual is, the better the support he or she can give to his or her children, and the less likely he or she is to be abused [32]. The more children there are, the greater the likelihood of physical abuse, financial abuse, and neglect. This may be because when they face support problems, the children tend to shirk their responsibilities and regard their elderly parent as a burden to the family, even beating and scolding him or her [19].

Moreover, we found that elderly people with a common-law spouse, those with a higher education level, those that frequently participate in social activities, and those with religious beliefs are less likely to suffer abuse (Table 2).

**Table 2:** Logistic regression analysis of factors associated with types of elder abuse

Variable	Physical abuse			Emotional abuse			Financial abuse			Neglect		
	B	S.E.	OR (95% *CI*)	B	SE	OR (95% *CI*)	B	SE	OR (95% *CI*)	B	SE	OR (95% *CI*)
Age												
65 ~ 69			1.00			1.00			1.00			1.00
70 ~ 74	0.33	0.26	1.07 (0.95–1.82)	0.16	0.19	1.21 (0.94–2.17)	0.01	0.12	1.12 (0.82–1.37)	0.18**	0.11	1.25 (0.35–2.82)
75~	0.27	0.13	1.12 (0.96–0.79)	0.27	0.35	1.18 (0.83–1.65)	0.18	0.21	1.36 (0.86–1.45)	0.13**	0.34	1.56 (0.67–3.54)
Gender												
Male			1.00			1.00			1.00			1.00
Female	0.28	0.17	1.06 (0.93–1.24)	0.31*	0.57	1.15 (0.76–1.86)	0.34	0.18	1.85 (0.54–2.87)	0.39**	0.78	1.87 (0.76–2.18)
Marital status												
Married			1.00			1.00			1.00			1.00
Divorced												
Widowed	0.34	0.16	1.28 (0.76–1.21)	0.36	0.31	1.79 (0.48–2.16)	0.15	0.45	1.65 (0.67–2.18)	0.42**	0.64	1.86 (0.56–3.15)
Unmarried												
Education level												
Illiterate			1.00			1.00			1.00			1.00
Primary school to high school	−0.15	0.23	0.96 (0.87–1.19)	−0.19	0.36	0.79 (0.43–1.55)	−0.21**	0.11	0.54 (0.12–2.13)	− 0.26*	0.21	0.82 (0.15–1.34)
University and above	− 0.26	0.37	0.95 (0.73–1.41)	− 0.24	0.56	0.86 (0.35–2.06)	−0.54**	0.64	0.83 (0.27–1.67)	−0.23*	0.37	0.43 (0.02–1.83)
Income												
Under 5000			1.00			1.00			1.00			1.00
5001 ~ 30,000	−0.48**	0.31	0.86 (0.65–1.48)	− 0.15*	0.43	0.46 (0.17–1.05)	− 0.31**	0.07	0.68 (0.34–1.48)	− 0.36**	0.43	0.76 (0.01–1.54)
30,001~	−0.36**	0.19	0.93 (0.75–1.38)	−0.49*	0.87	0.38 (0.12–1.21)	−0.24**	0.16	0.87 (0.37–1.39)	−0.48**	0.33	0.67 (0.13–1.69)
Number of children												
0			1.00			1.00			1.00			1.00
1 ~ 2	0.21*	0.06	1.12 (0.87–1.29)	0.16	0.76	1.34 (0.66–2.15)	0.11*	0.04	1.23 (0.87–3.18)	0.31*	0.53	1.52 (0.63–2.18)
3~	0.16*	0.13	1.08 (0.96–1.75)	0.53	0.29	1.76 (0.87–3.64)	0.34*	0.18	1.67 (0.54–1.65)	0.21*	0.44	1.55 (0.37–2.16)
Health condition												
1			1.00			1.00			1.00			1.00
2 ~ 3	−0.15	0.41	0.86 (0.73–1.45)	−0.27	0.23	0.49 (0.06–2.15)	−0.13	0.12	0.89 (0.18–1.33)	−0.36	0.74	0.85 (0.11–1.09)
4 ~ 5	−0.18	0.45	0.84 (0.71–1.42)	−0.25	0.91	0.73 (0.24–1.97)	−0.35	0.52	0.85 (0.27–1.73)	−0.53	0.08	0.77 (0.03–1.24)
Cognitive ability												
0 ~ 10			1.00			1.00			1.00			1.00
11 ~ 20	−0.26	0.17	0.76 (0.68–0.99)	− 0.18	0.14	0.86 (0.45–2.97)	− 0.01	0.01	0.97 (0.37–1.54)	− 0.32*	0.35	0.94 (0.18–1.27)
21 ~ 30	−0.18	0.34	0.89 (0.81–1.36)	−0.23	0.78	0.48 (0.05–1.65)	−0.08	0.12	0.79 (0.18–1.67)	−0.11*	0.29	0.92 (0.24–1.65)
Social frequency												
0 ~ 1			1.00			1.00			1.00			1.00
2 ~ 4	−0.15	0.23	0.98 (0.81–1.27)	− 0.26**	0.26	0.78 (0.31–1.54)	− 0.37**	0.08	0.68 (0.15–2.54)	− 0.21	0.34	0.89 (0.34–1.88)
5 ~ 7	−0.16	0.31	0.73 (0.48–1.15)	−0.73**	0.84	0.86 (0.21–1.89)	−0.17**	0.42	0.67 (0.11–1.93)	−0.33	0.18	0.83 (0.15–1.58)
Religious belief												
Yes			1.00			1.00			1.00			1.00
No	0.47	0.24	1.47 (1.01–1.96)	0.75**	0.65	2.54 (0.67–4.32)	0.28	0.17	1.35 (0.58–3.49)	−0.26**	0.64	0.77 (0.11–1.64)

*p < 0.05, **p < 0.01

## Discussion

This study enrolled elderly Chinese individuals as a sample during the COVID-19 outbreak and analyzed the prevalence of elder abuse and victim-related risk factors in this group. Comparing data from Women’s Federation and the National Bureau of Statistics, it was found that the prevalence of financial abuse during the epidemic was significantly higher than that in 2010 [23]. The epidemic may have affected the financial situation of the elderly individuals and their caregivers. During the epidemic, many factories and shops ceased operations, and a large number of workers were fired or forced to take leave. According to data released by the National Bureau of Statistics, in the first half of 2020, the per capita disposable income of urban residents in Hunan Province was 19,589 yuan, a year-on-year decrease of 50.8%. The per capita disposable income of rural residents was 7566 yuan, a year-on-year decrease of 50.9%. However, the consumer price index of Hunan Province rose by 3.5% year-on-year [3]. Economic pressure has reduced their support for their elderly parents, and even demanded money from their parents. The increase in neglect is likely related to social distancing measures, which significantly reduces the frequency with which caregivers visit the elderly. The government encourages people to stay in their own homes as much as possible. Many communities and villages have adopted closure measures, prohibiting people who are not in their own communities or villages from entering. These measures reduce the chance of face-to-face contact between caregivers and the elderly. In addition, the prevalence of different types of abuse are related. Individuals who have suffered physical abuse and financial abuse may also experience emotional abuse and neglect [20].

First, income is the only factor that had an important influence on the prevalence of the four types of abuse. In China, the income sources of the elderly mainly include minimum living allowance, retirement pension, property income (stocks, interest, rent, etc.), and labor income. The lower the income of an elderly individual was, the greater the likelihood of abuse. The lower the income of an elderly individual was, the greater the economic pressure was on their children as caregivers of the elderly individual. Therefore, we can say that economic pressure is the primary cause of abuse of the elderly [33]. This result confirms the explanatory effect of stress theory on elder abuse [34]. Lee et al. found that elderly people provide financial support and service support to their children in exchange for filial piety. The expectation of elderly parents for their children’s maintenance responsibilities is related to the amount of support they provide to their children [35]. During the epidemic, due to economic instability, adult children’s needs for income increased while the financial support that elderly parents could provide was relatively reduced. A large number of workers were fired or forced to take vacations, so they had more time to stay at home. On the one hand, the need for elderly individuals to look after children and take care of housework was decreased. On the other hand, the longer the time the elderly individual, adult children and grandchildren spend together, the more the possibility of conflict increases, leading to an increased risk of elder abuse [8, 9]. Furthermore, social distancing measures limit the opportunities for elderly individuals to interact with friends and obtain social support, which increases the risk of abuse to a certain extent [36, 37].

The study also found that as the number of children increases, elderly individuals are more likely to suffer physical abuse, financial abuse and neglect. In China, adult children taking care of elderly people in the family is a legal responsibility, not a choice [38]. When the number of children increases, the possibility for them to shirk their responsibilities also increases [19]. Finally, the regression results revealed that religious belief has reduces the likelihood of elderly abuse. This finding may be because religious doctrines generally call for being kind to others, especially elderly people.

The risk factors for elder abuse in China are quite different from those in other countries. We believe this is related to China’s old-age care model and traditional culture. At present, elderly people in China mainly rely on their children for support [23]. China’s old-age security system is not sound, and people pay attention to the improvement of economic conditions and ignore ideological education. The traditional ideology of respecting elderly individuals and traditional family ethics are relatively weak, which has led to frequent incidents of elder abuse [33]. Generally, the education level of an elderly individual is positively correlated with income; that is, the higher an individual’s educational background is, the higher his or her income. Moreover, factors such as health, cognitive function, and social participation are also related to income among elderly individuals. Therefore, we believe that the best course of action is to increase the income and social support of elderly people. Furthermore, the self-protection awareness of elderly individuals should be improved. We should help elderly individuals understand what elder abuse is, how to respond to abuse and what countermeasures to take [16].

This study has some limitations. First, due to limitations in data collection, caregivers were not included in the analysis. Second, because the study was based on cross-sectional data, it was impossible strictly to control for the temporal sequence of the independent variables and the dependent variables. In addition, the questionnaire mainly involved self-reports of elderly individuals, and we excluded people with mental and language disorders. Elderly individuals often conceal abuse for various reasons, so the survey data may have been biased. Therefore, in the future, multiregional and large-sample longitudinal studies are needed to obtain more reliable supporting evidence. Moreover, it is necessary not only to compare the changes in the prevalence of elder abuse but also to analyze the changes in the risk factors related to such abuse and to include abusers in the scope of the study.

## Data Availability

The datasets used and/or analyzed during the current study are available from the corresponding author upon reasonable request.

## Abbreviations

UN: United Nations
SE: Standard error
OR: Odds ratio
CI: Confidence interval
